# Astragaloside IV ameliorates 2,4,6-trinitrobenzene sulfonic acid (TNBS)-induced colitis implicating regulation of energy metabolism

**DOI:** 10.1038/srep41832

**Published:** 2017-02-02

**Authors:** Xu-Guang Jiang, Kai Sun, Yu-Ying Liu, Li Yan, Ming-Xia Wang, Jing-Yu Fan, Hong-Na Mu, Chong Li, Yuan-Yuan Chen, Chuan-She Wang, Jing-Yan Han

**Affiliations:** 1 Tasly Microcirculation Research Center, Peking University Health Science Center, Beijing, China; 2 Shandong college of Traditional Chinese Medicine, Yantai, Shandong, China; 3 Department of Integration of Chinese and Western Medicine, School of Basic Medical Sciences, Peking University, Beijing, China; 4 Key Laboratory of Microcirculation, State Administration of Traditional Chinese Medicine, Beijing 100191, China; 5 Key Laboratory of Stasis and Phlegm of State Administration of Traditional Chinese Medicine, Beijing 100191, China

## Abstract

Dysfunction of energy metabolism is involved in inflammatory bowel disease (IBD). This study was designed to investigate the potential of astragaloside IV (ASIV), an active ingredient of *Radix Astragalus*, to ameliorate colonic mucosal injury, with focusing on the implication of energy restoration in the underlying mechanism. Experimental colitis model was established in rats by injecting 2,4,6-trinitrobenzene sulfonic acid (TNBS) through anus. After 24 hours, ASIV was administrated once daily by gavage for 6 days. On day 1 and day 7, colon tissue was collected for macroscopic and histological examination, ELISA, Western blot and immunohistochemical analysis. TNBS impaired colonic mucosa with an injured epithelial architecture, increased inflammatory cell infiltration, and decreased colonic blood flow. Lgr5 positive cell number in crypt and β-catenin nuclear translocation were down-regulated by TNBS treatment. TNBS induced epithelial F-actin disruption and junctional protein degradation. Furthermore, adenosine triphosphate (ATP) content and ATP synthase subunit β expression in the colon tissue were significantly decreased after TNBS stimulation. All of the aforementioned alterations were relieved by ASIV post-treatment. The present study revealed that ASIV promoted mucosal healing process in TNBS-induced colitis, which was most likely attributed to regulating energy metabolism.

Inflammatory bowel disease (IBD), which includes ulcerative colitis (UC) and Crohn’s disease (CD), is an idiopathic disease characterized by chronic, relapsing inflammation and erosion as well as ulcer in the gastrointestinal tract with increased risk of developing colorectal cancer^1^,^2^. The pathophysiology and etiology of IBD have not been fully understood so far, though, repeated intestinal epithelial damage with disruption of mucosal barrier function has been recognized as one of the crucial pathogenic factor in IBD^3^,^4^. Thus, regenerating epithelial architecture to restore the normal function of colon has become a standard treatment goal in the management of IBD in clinic^5^,^6^. A series of clinical studies have demonstrated that complete regeneration of the intestinal mucosa predicts long-term remission^6^, while failure to achieve mucosal healing may predict a poor prognosis^7^. Accordingly, development of drugs essential for promoting recovery of the impaired mucosal epithelium has received increasing attention.

The intestinal epithelium is one of the tissues that are most capable of self-renewal in the body, which is supported by proliferative resident intestinal stem cells (ISC). ISC are located close to the crypt base and produce vigorously proliferating progenitors called transit-amplifying cells, which continuously move upward as coherent columns to replace the died and shed epithelial cells^8^. However, in a variety of human diseases, including IBD as well as colon cancer, this renewal process is dysregulated and the homeostasis in the intestinal epithelium is disturbed. Researches have been conducted to restore the homeostasis of intestinal epithelium after injury^9^,^10^, but none of them has been translated to clinic.

Dysfunction of energy metabolism has been shown to be implicated in the pathogenesis of IBD^11^. Inflammation reaction, such as neutrophils infiltration and phagocytosis, increases the demand of energy. On the other hand, inflammation-induced abnormal microvasculature causes a decreased supply of nutrient and oxygen that further aggravates the energy deficiency. This energy deficiency contributes to the disruption of mucosal epithelium barrier found in IBD, which is regulated by interendothelial cell junctions and F-actin filaments, both of which need energy to maintain integrity^12^,^13^,^14^. In addition, increasing evidence suggests that restoration of injured epithelium in IBD is an energy consuming process^15^. Therefore, regulating energy metabolism is anticipated as a promising strategy for IBD therapy.

Astragalosides IV (ASIV, Fig. 1A) is one of the major active ingredient of *Radix Astragalus* (RA), a well-known Chinese medicine with tonic property. RA has been used to promote burn wound healing with efficiency. Previous study demonstrated that RA extracts boost healing of open cutaneous wounds by suppressing inflammation and stimulating basal cell growth^16^. RA saponins were found to be capable of stimulating re-epithelization and neovascularization in skin lesions^17^,^18^,^19^. In addition, a recently study showed that VEGF- and Akt-dependent signaling pathways are involved in the pro-angiogenic effect of ASIV^20^. Our previous study has proved that Huang-Qi-Jian-Zhong pellet, a formula containing RA as a major component, accelerates the recovery of colonic injury in experimental colitis animals^21^. Particularly, ASIV was demonstrated to regulate myocardial adenosine triphosphate (ATP) synthase activity by restoring ATP synthase subunit δ (ATP5D) expression after ischemia and reperfusion injury^22^. Based on these data, we speculated that ASIV may have a potential for mucosal healing in IBD with implication of energy regulation. The present study was designed to test this speculation by using trinitrobenzene sulfonic acid (TNBS)-induced colitis model.

## Results

### ASIV reduces TNBS-induced colitis in rats in a dose- and time-dependent manner

**Figure 1:**
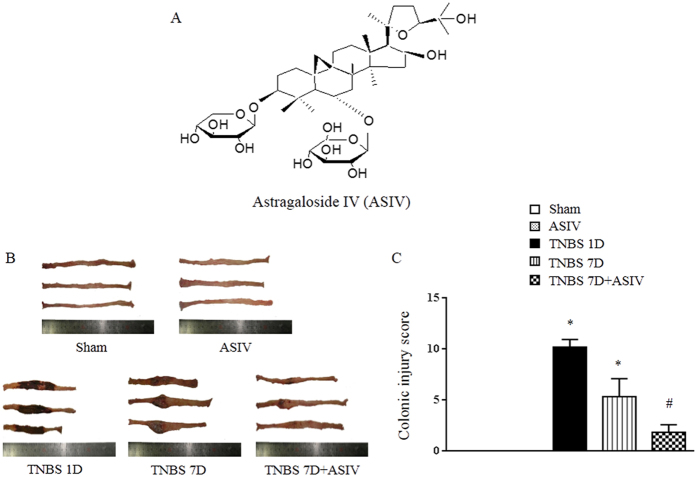
ASIV reduces TNBS-induced colitis in rats. (**A**) Chemical structure of ASIV. (**B**) Representative image of colon in different groups. (**C**) Macroscopic injury score. Sham: sham group; ASIV: ASIV alone group; TNBS 1D: TNBS treatment for 1 day group; TNBS 7D: TNBS treatment for 1 days followed by saline treatment for 6 days group; TNBS 7D + ASIV: TNBS treatment for 1 day followed by ASIV treatment for 6 days group. Data are mean ± SEM (N = 8). *p < 0.05 vs. Sham group, #*p* < 0.05 vs. TNBS 7D group.

We first evaluated the efficacy of ASIV in coping with colitis in rats, which were challenged by TNBS for 24 hours followed by treatment with ASIV for 6 days. As shown in Fig. 1B,C and Supplement Figure 1, TNBS challenge for 24 hours evoked obvious damage in colon, including atrophy, hyperemia, edema, erosion and degeneration, which sustained until day 7, though to a less extent, if without intervention. Strikingly, treatment with ASIV at 10 mg/kg for 6 days promoted colitis recovery, exhibiting only a slight hyperemia and inflammation. In addition, we found no significant effects of ASIV on the mucosal damages at the dose of 5 mg/kg 7 days after TNBS stimulation, nor for ASIV at 5 mg/kg or 10 mg/kg 4 days after TNBS stimulation (Supplement Figure 1), suggesting the improvement effects of ASIV on TNBS-induced mucosal injury was dose- and time-dependent. Meanwhile, the animals in ASIV only group did not show any change in colon, as compared with that in sham group (Fig. 1B and C).

### Histological evidence for the beneficial role of ASIV in TNBS-induced colitis in rat

**Figure 2:**
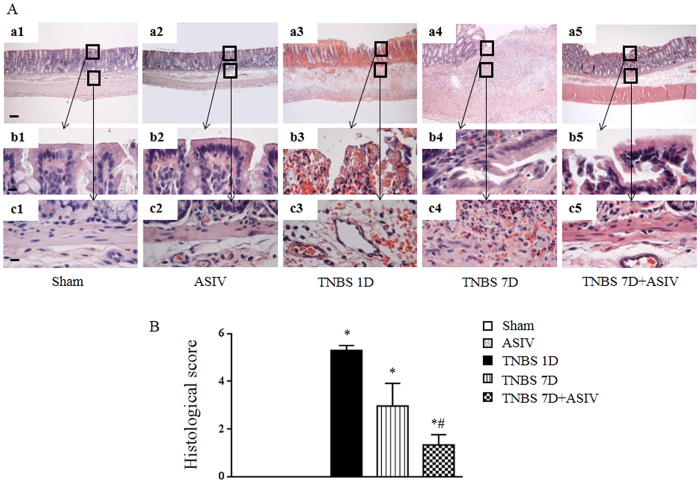
ASIV suppresses histological injury in TNBS-induced colitis in rats. (**A**) Representative images of HE staining of colon tissue from different groups (a1-a5). Bar = 100 μm. The area within the rectangle in each picture is enlarged and presented below, correspondingly, to display the mucosa (b1-b5) and submucosa (c1-c5) in each group. Bar = 10 μm. (**B**) Colonic histological score. Sham: sham group; ASIV: ASIV alone group; TNBS 1D: TNBS treatment for 1 day group; TNBS 7D: TNBS treatment for 1 days followed by saline treatment for 6 days group; TNBS 7D + ASIV: TNBS treatment for 1 day followed by ASIV treatment for 6 days group. Data are mean ± SEM (N = 8). *p < 0.05 vs. Sham group, #*p* < 0.05 vs. TNBS 7D group.

Observation by microscopy provided some details for the beneficial effect of ASIV on colitis caused by TNBS. Figure 2A shows the images of HE-staining colon tissue sections from different groups. Compared to Sham and ASIV alone group, an apparent injury was found in TNBS 1D group, including mucosal epithelial necrosis, hyperemia, edema, and inflammatory cell infiltration (Fig. 2A, a3-c3). A similar, but less obvious, structural abnormalities in colonic mucosa was noticed in TNBS 7D group, with remained hyperemia, inflammation and microvascular occlusion in the submucosa (Fig. 2A, a4-c4). As expected, TNBS-induced alterations in rat colonic mucosa were significantly restored by treatment with ASIV, as evidenced by regeneration of crypt and villus integrity, increased vascular density and deceased erosion (Fig. 2A, a5-c5). These results were confirmed by a quantitative assessment of the morphological manifestation (Fig. 2B).

Immunohistochemistry of colon tissue was conducted for assessment of MPO and CD68, which are the biomarker of infiltrating neutrophils and monocytes, respectively. As shown in Supplement Figure 2A,B and C, the MPO expression was significantly elevated in TNBS 1D group, which became reduced in TNBS 7D group. While CD68 expression increased with time. ASIV administration for 6 days relieved the TNBS-caused expression of both MPO and CD68.

### ASIV promotes epithelium reconstitution in TNBS-treated rat colon

Immunohistochemistry was conducted to assess the expression of Ki67 in colon epithelium (Supplement Figure 3A and B). Compared with Sham and ASIV alone group, less Ki67-positive cells were found in TNBS 1D group. The number of Ki67-positive cells increased in TNBS 7D group, implying a spontaneous recovery of the injured epithelium. ASIV administration resulted in a further increase in Ki67 positive cells. Western blot analysis showed that the expression of E-cadherin, a marker of epithelial cells, decreased in TNBS 1D group, but became recovered in TNBS 7D group (Supplement Figure 3C), which was significantly accelerated by ASIV treatment.

### ASIV increases the blood flow in TNBS-induced rat colon

**Figure 3:**
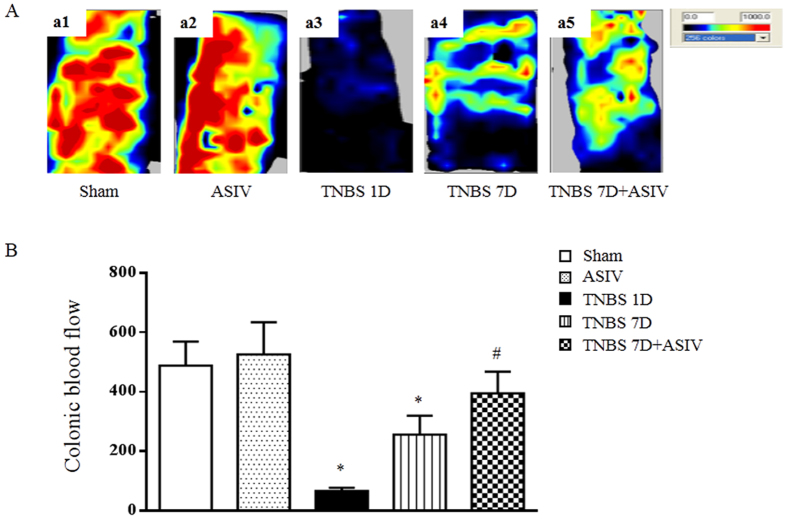
ASIV increases blood flow in TNBS-induced colitis in rats. (**A**) Representative images of colonic blood flow in each group. The magnitude of colonic blood flow is represented by different colors, with blue to red denoting low to high. (**B**) Quantitative analysis of colonic blood flow in different groups. Sham: sham group; ASIV: ASIV alone group; TNBS 1D: TNBS treatment for 1 day group; TNBS 7D: TNBS treatment for 1 days followed by saline treatment for 6 days group; TNBS 7D + ASIV: TNBS treatment for 1 day followed by ASIV treatment for 6 days group. Data are mean ± SEM (N = 8). *p < 0.05 vs. Sham group, #*p* < 0.05 vs. TNBS 7D group.

As a source of oxygen and nutrient, blood flow plays a critical role in inflammation and injury recovery. Thus, blood flow in colon was determined by laser Doppler perfusion in different groups, and the representative images and quantitative results are shown in Fig. 3A and B, respectively. As expected, the mucosal blood flow in TNBS 1D group showed a dramatic reduction compared with Sham group, which, albeit recovered to some extent, was still much lower in TNBS 7D group than Sham and ASIV alone group. Impressively, ASIV administration for 6 days significantly improved the mucosal blood flow compared to the TNBS 7D group.

### ASIV increases intestinal stem cell number in mucosal crypt of TNBS-stimulated rat colon

**Figure 4:**
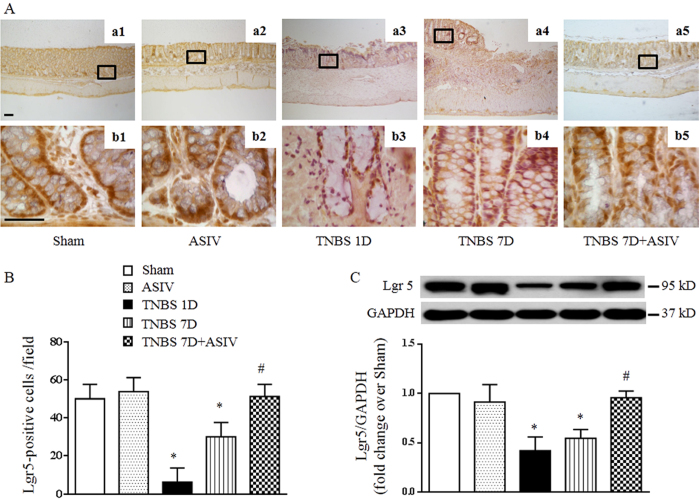
ASIV increases intestinal stem cell number in crypt of TNBS- challenged rat colon. (**A**) Representative images of immunohistochemistry staining for Lgr5 (brown) of colon tissue from different groups (a1-a5). Bar = 100 μm. The area within the rectangle in each picture is enlarged and presented below correspondingly (b1-b5, Bar = 50 μm). (**B**) Quantification analysis of Lgr5-positive cells in different groups (N = 8). (**C**) Representative Western blots and statistical analysis of Lgr5 in colon tissue in different groups (N = 5). Sham: sham group; ASIV: ASIV alone group; TNBS 1D: TNBS treatment for 1 day group; TNBS 7D: TNBS treatment for 1 days followed by saline treatment for 6 days group; TNBS 7D + ASIV: TNBS treatment for 1 day followed by ASIV treatment for 6 days group. Data are mean ± SEM. *p < 0.05 vs. Sham group, #*p* < 0.05 vs. TNBS 7D group.

The intestine epithelium has a considerable capacity to repair after damage, mainly depending on its continuous replenishment fueled by resident stem cells, which reside at the crypt base and give rise to all the differentiated cell types. Immunohistochemistry was performed in the present study to assess the expression of Lgr5, a biomarker of stem cells^23^, in different groups. The results demonstrated that under normal circumstances, the positive staining of Lgr5 at the crypt base was obvious (Fig. 4A, a1, b1, a2, b2). On the contrary, Lgr5 staining decreased remarkably in TNBS 1D group (Fig. 4A, a3, b3), which recovered to some degree in TNBS 7D group, though, but was still lower than Sham and ASIV alone group (Fig. 4A, a4, b4). Excitingly, Lgr5 staining in ASIV treatment group increased to a level close to normal (Fig. 4A, a5, b5 and B). Consistent with this result, Western blot analysis showed that TNBS induced a significant reduction of Lgr5 expression in colon tissue, which was ameliorated by ASIV treatment (Fig. 4C).

**Figure 5:**
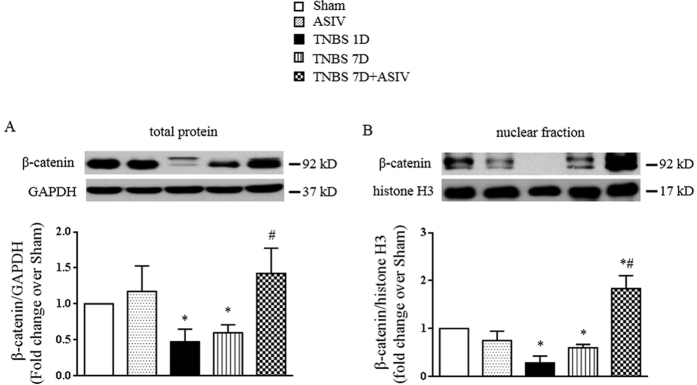
ASIV stimulates β-catenin nuclear translocation in TNBS-challenged rat colon. (**A**) and (**B**) Representative Western blots and statistical analysis of the expression of total and nuclear faction of β-catenin in colon tissues from different groups, respectively. All blots are representative of 5 separate experiments. Sham: sham group; ASIV: ASIV alone group; TNBS 1D: TNBS treatment for 1 day group; TNBS 7D: TNBS treatment for 1 days followed by saline treatment for 6 days group; TNBS 7D + ASIV: TNBS treatment for 1 day followed by ASIV treatment for 6 days group. Data are mean ± SEM. *p < 0.05 vs. Sham group, #*p* < 0.05 vs. TNBS 7D group.

As Lgr5 is a Wnt target gene^24^, we next examined the changes of the key molecule in the Wnt signaling pathway after TNBS stimulation and ASIV administration. The expression and nuclear translocation of β-catenin, a key downstream transcriptional factor of Wnt in regulating Lgr5 expression, varied among groups in a similar manner (Fig. 5A and B). These data suggested that ASIV stimulated intestinal stem cell driving epithelium regeneration, which might be correlated with its stimulation of β-catenin nuclear translocation.

### ASIV ameliorates TNBS-induced actin filaments disruption and tight junction protein degradation

**Figure 6:**
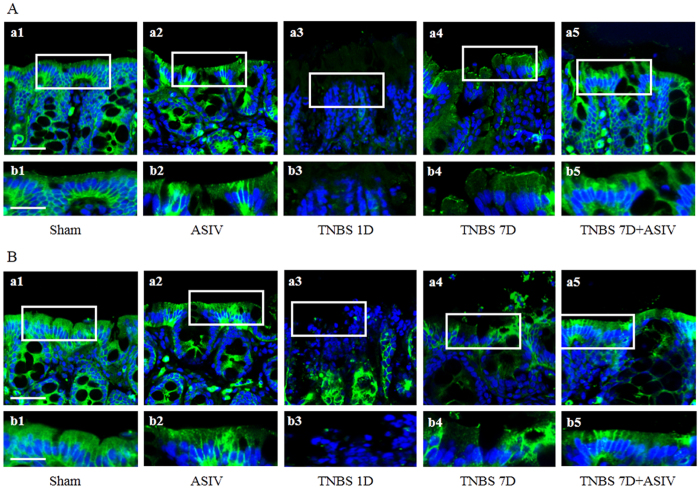
ASIV ameliorates tight junction protein disarrangement in TNBS-challenged colon. (**A**) and (**B**) Representative immunofluorescence confocal images (a1-a5, Bar = 50 μm) for Occludin (**A**), green) and claudin-5 (**B**), green) of colonic epithelium from different groups, respectively. The area within the rectangle in each picture is enlarged and presented below, correspondingly (b1-b5, Bar = 25 μm). Sham: sham group; ASIV: ASIV alone group; TNBS 1D: TNBS treatment for 1 day group; TNBS 7D: TNBS treatment for 1 days followed by saline treatment for 6 days group; TNBS 7D + ASIV: TNBS treatment for 1 day followed by ASIV treatment for 6 days group.

Epithelial barrier integrity is of pivotal impotence for normal function of intestine, which is regulated by intercellular junctions and disrupts in IBD. To assess the role of ASIV in restoring the disrupted epithelial barrier in TNBS-induced colitis, an immunofluorescence staining of two tight junction protein, occludin and claudin-5, in mucosal epithelium was performed in the present study for different groups. In Sham and ASIV alone group, both occludin (Fig. 6A, a1, b1 and a2, b2) and claudin-5 (Fig. 6B, a1, b1 and a2, b2) in the mucosal epithelium were abundant and well-organized. However, expression of occludin (Fig. 6A, a3, b3) and claudin-5 (Fig. 6B, a3, b3) in epithelial cells revealed a noticeable decrease in TNBS 1D group. In TNBS 7D group, the expression of occludin (Fig. 6A, a3, b3) and claudin-5 (Fig. 6B, a3, b3) increased as compared to that in TNBS 1D group, while the distribution was disordered (Fig. 6A, a4, b4; and B, a4, b4). Interestingly, ASIV treatment for 6 day significantly promoted the recovery of occludin and claudin-5 in epithelial cells (Fig. 6A, a5, b5; and B, a5, b5).

**Figure 7:**
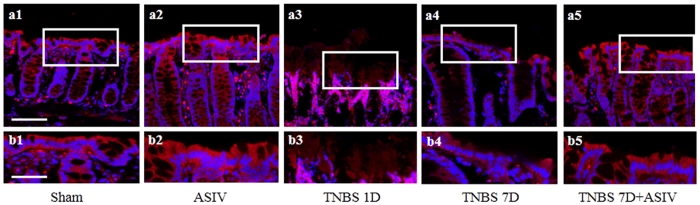
ASIV ameliorates TNBS-induced actin filaments disruption in rat colonic mucosal epithelium. (a1-a5) Representative immunofluorescence confocal images for F-actin of colonic epithelium from different groups. Bar = 50 μm. The area within the rectangle in each picture is enlarged and presented below correspondingly (b1-b5, Bar = 25 μm). Sham: sham group; ASIV: ASIV alone group; TNBS 1D: TNBS treatment for 1 day group; TNBS 7D: TNBS treatment for 1 days followed by saline treatment for 6 days group; TNBS 7D + ASIV: TNBS treatment for 1 day followed by ASIV treatment for 6 days group. Data are mean ± SEM. *p < 0.05 vs. Sham group, #*p* < 0.05 vs. TNBS 7D group.

Actin cytoskeleton is known to interact with the tight junction and modulate tight junction permeability. We then examined the F-actin structure in the epithelium from various groups. The representative confocal microscopic images are displayed in Fig. 7, wherein Sham and ASIV alone group revealed a normal F-actin structure, while a dramatic disruption of actin filaments was observed 1 day or 7 days after TNBS stimulation (Fig. 7, a3, b3 and a4, b4). In contrast, this actin depolymerization was protected evidently by treatment with ASIV for 6 days (Fig. 7, a5, b5).

### ASIV regulates colonic energy status

**Figure 8:**
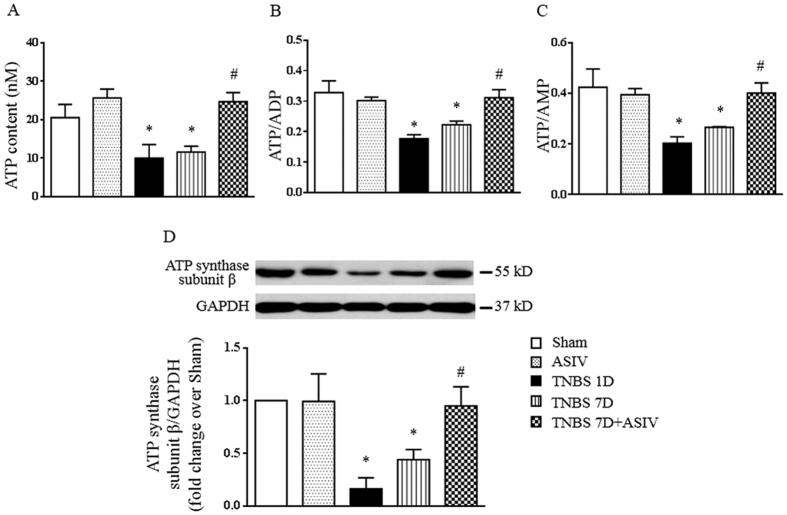
ASIV increases ATP content and ATP synthase subunit β expression in TNBS-challenged rat colon. (**A**) The concentration of ATP in colon tissues from different groups. (**B**) and (**C**) The ratio of ATP/ADP and ATP/AMP, respectively, in different groups. (**D**) Representative Western blots and statistical analysis of ATP synthase subunit β in colon tissues from different groups. Sham: sham group; ASIV: ASIV alone group; TNBS 1D: TNBS treatment for 1 day group; TNBS 7D: TNBS treatment for 1 days followed by saline treatment for 6 days group; TNBS 7D + ASIV: TNBS treatment for 1 day followed by ASIV treatment for 6 days group. Data are mean ± SEM (N = 5). *p < 0.05 vs. Sham group, #*p* < 0.05 vs. TNBS 7D group.

As ATP is indispensable for maintaining the integrity of actin filament and intercellular junctions, as well as necessary for the recovery of injured epithelium, the ATP, ADP and AMP content was thus analyzed in the present study by ELISA. As shown in Fig. 8A,B and C, compared with Sham and ASIV alone group, a significant decrease was observed in ATP content, the ratio of ATP/ADP and ATP/AMP 1 day and 7 days after TNBS stimulation. This TNBS-induced decrease was restored significantly by treatment with ASIV.

To gain insight into the mechanism thereby ASIV upregulated ATP synthesis, we next determined the expression of ATP synthase catalytic subunit (ATP synthase subunit β), the key ATP synthase subunit, in each group by Western blot. The result showed that the expression of ATP synthase subunit β in TNBS-induced rat colitis decreased significantly on day 1 and day 7, which was significantly ameliorated by ASIV treatment (Fig. 8D). These data suggested that ASIV improved colonic energy metabolism via upregulation of ATP synthase subunit β.

## Discussion

TNBS is widely used as a stimulus to establish animal model for IBD. Consistent with others, we observed in the present study an obvious injury in colon tissue 1 day after TNBS challenge manifesting mucosal epithelium damage, inflammatory cell infiltration and reduced colon blood flow. Of notice, all the insults became less prominent 7 days after TNBS administration, implying the presence of a spontaneous recovery process. Interestingly, treatment with ASIV for 6 days significantly accelerated this recovery process leading to a much better outcome. These results provided evidence for the potential of ASIV in coping with IBD.

The result of the present study suggested that the colon tissue has a mechanism to repair the injured epithelium. In the present setting, it is not known how far this recovery process can move forward without intervention. Nevertheless, ASIV treatment significantly enhanced the repair progressing. The capacity of ASIV to aid in promotion of epithelium regeneration has been suggested by the researches of *Radix Astragalus,* the herb that contains ASIV as an ingredient, which has been shown to stimulate re-epithelization and used for healing of cutaneous wounds or ulcers. The result of the present study indicated that it is the ASIV in *Radix Astragalus* that is responsible for the potential of this herb in this respect.

Two processes may contribute to the repair of injured epithelium. One is the proliferation of the matured epithelial cells. The other is the differentiation of resident stem cell population in the crypt, which normally continuously renews the epithelial cells lining the colon mucosal surface. The results of the present study revealed that both processes were dysregulated after TNBS stimulation, as evidenced by the decrease in Ki67 staining of mucosal epithelium and the expression of Lgr5 and β-catenin, in which Ki67 represents epithelial cells undergoing proliferation, while Lgr5 is a biomarker of stem cell the expression of which is regulated by β-catenin nuclear translocation^24^. Noticeably, ASIV treatment boosted the proliferation of epithelial cells as well as increased the number of stem cells. The mechanism thereby ASIV modulated the proliferation of epithelial cells and of stem cells is at present unknown. However, the fact that ASIV interfered in both events implies that ASIV may act some target that governs both processes. A likely candidate for this target is ATP synthase.

The importance of ATP synthase and ATP in regulation of cell proliferation has been noticed previously in some studies. It was reported that ATP controls cell cycle and induces cell proliferation by promoting late developing progenitors to progress from G1 to S phase of cell cycle^25^. Barbosa and colleges demonstrated that ATP regulates differentiation of hematopoietic stem cell^26^. A resent publication showed that ATP synthase promotes germ cell differentiation independent of oxidative phosphorylation^27^. It is thus predicated that a normally functioning ATP synthase and improved ATP supply may help restore the injured mucosal epithelium in IBD. Furthermore, an improved energy metabolism is needed as well for maintaining mucosal epithelium barrier, particularly the integrity of F-actin and junctions proteins, the major determinants of epithelium barrier, which has critical role in the function of colonic mucosa. On the other hand, we have previously showed the ability of ASIV to restore ATP synthase activity in myocardium after ischemia and reperfusion^22^. These data, together with the finding in the present study that ASIV treatment elevated the ATP level and increased the expression of ATP synthase subunit β, meanwhile, prevented F-actin depolymerization and tight junction protein downregulation, highly suggest ATP synthase as the target for ASIV action. ASIV relieves the decreased ATP synthase subunit improving ATP supply, which suggests that ASIV attenuates the impaired ATP synthase subunit by impacting on some link upstream ATP synthase. Nevertheless, the mechanism for the beneficial role of ASIV requires further study.

In conclusion, the present study demonstrated that ASIV has a therapeutic effect on colonic mucosal injury, accelerating epithelial cell proliferation, provoking stem cell growth, and restoring the mucosal barrier relevant proteins, which is most likely attributable to its potential to attenuate the impaired ATP synthase. These data provide an alternative option for the development of new medications for patients with IBD by restoring energy supply to promote mucosal injury recovery.

## Materials and Methods

### Animals

Male Sprague-Dawley (SD) rats weighing 180 to 200 g were purchased from the Animal Center of Peking University Health Science Center (Beijing, certificate no. SCXK 2006-0008). The animals were housed in cages at 22 ± 2 °C and humidity of 40 ± 5% in a 12-hour light/dark cycle, and received standard diet and water *ad libitum*. The rats were fasted for 12 hours before experiment but allowed free access to water. The experimental procedures were carried out in accordance with the European commission guidelines (2010/63/EU). All animals were handled according to the guidelines of the Peking University Animal Research Committee. The protocols were approved by the Committee on the Ethics of Animal Experiments of the Health Science Center of Peking University (LA2011-38).

### Drugs and reagents

ASIV of high purity (>98% by HPLC) was obtained from Feng-Shan-Jian Medicine Research Co. Ltd. (Kunming, Yunnan, China). It was dissolved in 0.5% sodium carboxymethyl cellulose (CMC) at a concentration of 0.5 mg/mL before experiment. 5% TNBS solution was purchased from Sigma (St. Louis, MO, USA). CMC was obtained from Tianjin Run-Sheng Cellulose Technology Co. Ltd (Tianjin, China).

### IBD model

IBD was induced by TNBS in rats as reported previously^21^. Briefly, 5% TNBS solution was well mixed with equal volume of 30% ethanol solution before use. After anesthetized with pentobarbital sodium (0.1 g/kg body weight, i.p.), rat was administrated with TNBS at a dose of 100 mg/kg by injection into the colon 8 cm proximal to the anus using a polyvinyl catheter 2 mm in diameter. The rat was maintained in a head-down position for 15 minutes.

### Experimental groups

The rats were randomly divided into 5 groups, 16 animals in each. In the TNBS groups, the rats were given TNBS via clyster and sacrificed 24 hours thereafter (TNBS 1D). Alternatively, 24 hours after TNBS administration, rats received CMC solution by gavage (2 mL/kg) every 12 hours for another 6 days (TNBS 7D). In the ASIV treatment group (TNBS 7D + ASIV), rats were administrated with ASIV (10 mg/kg) by gavage starting from 24 hours after TNBS administration, twice a day, for 6 days. In the sham group (Sham) and ASIV alone group (ASIV), the TNBS was replaced by saline, and the same volume of CMC solution or ASIV (10 mg/kg) was infused in Sham or ASIV group for 6 days, respectively.

### Macroscopic evaluation

Rat was sacrificed under anesthesia and colon from cecum to anus was removed immediately along the mesenteric border, opened longitudinally, its content was cleaned with 4 °C saline. The colon was blotted with filter paper and laid flat on the paper for measurement of length and evaluation of injury.

The scoring for colonic macroscopic damage was evaluated using a criterion described by Butzner *et al*.^28^, in which 0 score: normal appearance; 1 score: focal hyperemia, no ulcers; 2 scores: ulceration without hyperemia or bowel wall thickening; 3 scores: ulceration with inflammation at one site; 4 scores: ≥ two sites of ulceration and inflammation; 5 scores: major sites of damage extending >1 cm along the length of the colon; 6–10 scores: damage extended to >2 cm along the length of the colon, increasing the score by one for each additional cm of damage.

Immediately after the scoring colon was weighed, and colon weight index (colonic weight/body weight × 100%) was calculated.

### Histological evaluation

A colon segment 8 cm proximal to anus was excised, fixed in 4% paraformaldehyde for 24 hours, dehydrated and embedded in paraffin. Five μm thick longitudinal paraffin sections were prepared, mounted on glass slides, and stained with hematoxylin and eosin (HE). The sections were observed with a light microscope (Nikon 90i; Nikon, France) and the damages were assessed both in a blind manner by two investigators according to a modified histological grading scale, which takes into consideration both inflammatory cell infiltration and tissue damage. As such, the inflammatory cell infiltration was scored as follows: 0 = no infiltration; 1 = increased number of inflammatory cells in the lamina propria; 2 = inflammatory cells extending into the submucosa; and 3 = transmural inflammatory cell infiltration. The tissue damage was scored as follows: 0 = no mucosal damage; 1 = discrete epithelial lesions; 2 = erosions or focal ulcerations; and 3 = severe mucosal damage with extensive ulceration extending into the bowel wall^29^.

### Colon blood flow assessment

Colonic blood flow was assessed by a Laser Doppler perfusion image system (PeriScan PIM3 System; Perimed, Stockholm, Sweden), as described previously^21^. Briefly, an incision was made through abdominal wall to expose peritoneal cavity, and a colon segment 6 to 8 cm proximal to anus was exposed. A computer-controlled optical scanner directed a low-powered He-Ne laser beam over the exposed colon, while epicolic tissues were covered with black soft leather. A color-coded image denoting specific relative perfusion level was displayed on a video monitor, and all images were evaluated with the software LDPIwin 3.1 (PeriScan PIM3 System; Perimed). The magnitude of blood flow was represented by different colors, with blue to red denoting low to high. Results were expressed as percentages of the baseline.

### Immunohistochemistry staining

Sections were deparaffinized by using xylene and graded ethanol. Endogenous peroxidase was blocked by incubating the sections in 3% H_2_O_2_-methanol at room temperature for 30 minutes. After washing in phosphate buffered saline (PBS), slides were incubated with 5% normal goat serum in PBS at 37 °C for 30 minutes to prevent nonspecific staining. The slides were then incubated with primary antibodies anti-Lgr5 (1: 200, Abcam, Cambridge, MA, USA) and anti-Ki-67 (1: 400, Abcam, Cambridge, MA, USA) diluted in PBS with 1% bovine serum albumin overnight at 4 °C. Specific binding was detected by incubation with horseradish peroxidase (HRP)-conjugated secondary antibody (ZSGB-BIO, Beijing, China) and revealed with the 3,3′-diaminobenzidine (DAB) substrate Kit. Myeloperoxidase (MPO) and CD68 were detected with double staining method in the same sections. In brief, section was incubated with rabbit anti-MPO (1: 200, Santa Cruz, CA, USA) and mouse anti-CD68 (1: 300, Abcam, Cambridge, MA, USA) diluted in PBS with 1% bovine serum albumin overnight at 4 °C. Specific binding was detected by incubation with HRP- and alkaline phosphatase -conjugated secondary antibody against rabbit and mouse, respectively (ZSGB-BIO, Beijing, China). Slides were counterstained with hematoxylin and mounted. Normal serum substituted for the primary antibodies served as control.

All sections were reviewed independently by two investigators using Image-pro plus 6.0 software. Five visual fields were selected from each section for analysis of the protein expression, and the mean density or the mean numbers of positive cell were determined.

### Immunofluorescence staining and confocal microscopy

The animals under anesthesia were infused via the left ventricle with 4% paraformaldehyde in 0.01 M PBS (pH 7.4). Colon segment 5 cm proximal to anus was removed and post-fixed with the same fixative for 12 hours, and cryoprotected in 30% sucrose in PBS for at least 24 hours at 4 °C. The colon with mucosal injure was sliced in 10 μm thick using a cryostat (CM1800; Leica, Bensheim, Germany). To evaluate the cell junction in colonic mucosa, immunofluorescence staining of occludin and claudin-5 was performed. For this purpose, slices were incubated with the following primary antibodies overnight at 4 °C: mouse anti-claudin-5 (1: 100, Invitrogen, Camarillo, CA, USA), mouse anti-occludin (1: 50, Invitrogen, Camarillo, CA, USA). After washing, sections were incubated with dylight 549-labeled goat anti-mouse IgG (KPL, Gaithersburg, MD, USA) for 2 hours at room temperature. Hoechst 33342 (BD Biosciences Pharmingen, San Jose, CA, USA) was applied to stain nucleus. F-actin in colonic tissues was stained with phalloidin (1: 40, Abcam, Cambridge, UK). All sections were photographed under a laser scanning confocal microscope (TCS SP5, Leica, Mannheim, Germany).

### Assessment of energy metabolism

The colon segment 8 cm proximal to anus was excised, quickly frozen in liquid nitrogen, and stored at −80 °C for a maximum of one week before use. The whole protein of the tissues was extracted with a protein extraction kit (Applygen Technologies, Beijing, China), according to manufacturer’s instruction. The content of ATP, ADP and AMP in colon was assessed with ELISA by microplate reader (MULTISKAN MK3, Thermo, CA, USA), according to the manufacturer’s instructions^22^.

### Western blot

The cytoplasmic, nuclear and membrane protein was extracted, respectively, by Nucl-Cyto-Mem Preparation Kit (Applygen Technologies, Beijing, China), according to the manufacture’s instruction. The concentration of whole protein was determined with a BCA protein assay kit (Applygen Technologies). After separated on 10% SDS-PAGE, the proteins were transferred to polyvinylidene difluoride membrane. Following blocking and rinsing with TBS-Tween (TBST), the membrane with target proteins was cut and incubated overnight at 4 °C with antibodies, respectively, against ATP Synthase subunit β (1: 200, Abcam, Cambridge, UK), Lgr5 (1: 1000, Cell Signaling Technology, Beverly, MA, USA), E-Cadherin (1: 1000, Abcam, Cambridge, UK), and β-catenin (1: 1000, Abcam, Cambridge, UK). The GAPDH (1: 5000, Cell Signaling Technology, Beverly, MA, USA), and histone H3 (1: 1000, Cell Signaling Technology, Beverly, MA, USA) were applied as loading control for cytoplasm and nucleus protein, respectively. After rinsing with TBST, the membranes were incubated with secondary antibody (Cell Signaling Technology, Beverly, MA, USA) for 1 hour at room temperature. The blots were developed using the SuperEnhanced Chemiluminescence detection kit (Applygen Technologies Inc., Beijing, China), and the protein bands were visualized after exposure of the membranes to Kodak X-ray film. The intensity of each band was evaluated with Quantity One software (Bio-Rad, CA, USA). For statistical analysis, the intensity ratio of target protein in each group to loading control in the same lane was calculated and presented as the fold change over Sham group^30^,^31^.

### Statistical Analysis

Results were expressed as mean ± SEM. Statistical analysis of data was performed by ANOVA for group followed by Tukey test for multiple-comparison. All statistical analyses were performed using GraphPad Prism 5 software. A value of *p* < 0.05 was considered statistically significant.

## Additional Information

**How to cite this article**: Jiang, X.-G. *et al*. Astragaloside IV ameliorates 2,4,6-trinitrobenzene sulfonic acid (TNBS)-induced colitis implicating regulation of energy metabolism. *Sci. Rep.* **7**, 41832; doi: 10.1038/srep41832 (2017).

**Publisher's note:** Springer Nature remains neutral with regard to jurisdictional claims in published maps and institutional affiliations.

## Supplementary Material

Supplement Information
