# Supplementary material for: Astragaloside IV ameliorates 2,4,6-trinitrobenzene sulfonic acid (TNBS)-induced colitis implicating regulation of energy metabolism
Source: Sci Rep. 2017 Feb 2;7:41832. doi: 10.1038/srep41832 (PMC5288804; doi:10.1038/srep41832)
Supplement: Supplement Information [file srep41832-s1.pdf]

Astragaloside IV ameliorates 2,4,6-trinitrobenzene sulfonic acid (TNBS)-induced colitis implicating regulation of energy metabolism

Xu-Guang Jiang [1,2,¶], Kai Sun [1,3,4,5,¶], Yu-Ying Liu [1,4,5], Li Yan [1,4,5], Ming-Xia Wang [1,4,5], Jing-Yu Fan [1,4,5], Hong-Na Mu [1,4,5], Chong Li [1,4,5], Yuan-Yuan Chen [1,4,5], Chuan-She Wang [1,3,4,5], Jing-Yan Han [1,3,4,5,*]

1. Tasly Microcirculation Research Center, Peking University Health Science Center, Beijing, China
2. Shandong college of Traditional Chinese Medicine, Yantai, Shandong, China
3. Department of Integration of Chinese and Western Medicine, School of Basic Medical Sciences, Peking University, Beijing, China
4. Key Laboratory of Microcirculation, State Administration of Traditional Chinese Medicine, Beijing 100191, China
5. Key Laboratory of Stasis and Phlegm of State Administration of Traditional Chinese Medicine, Beijing 100191, China

¶Co-first author of this paper and contribute equally

Supplement Figure 1

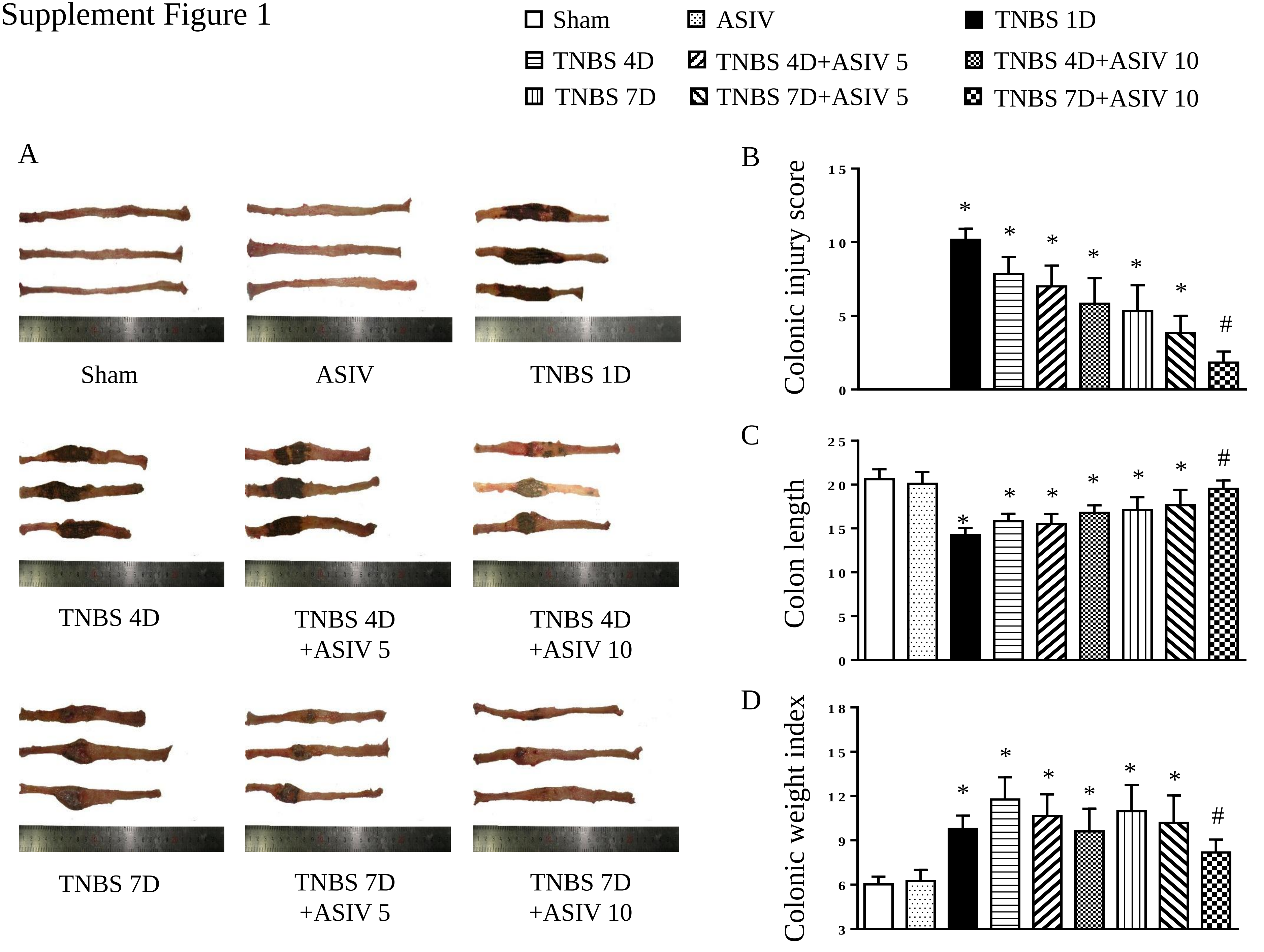

Supplement Figure 2

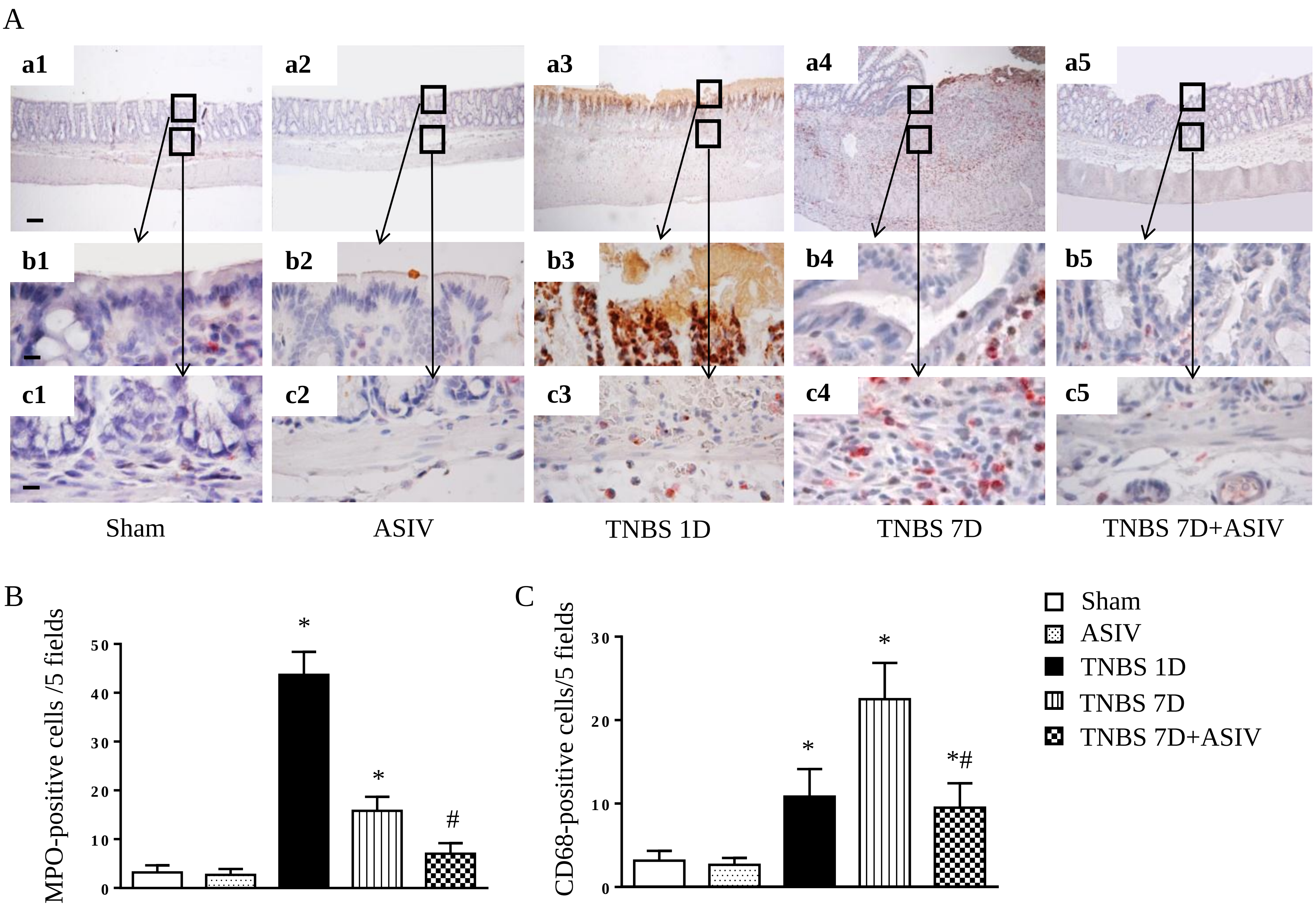

Supplement Figure 3

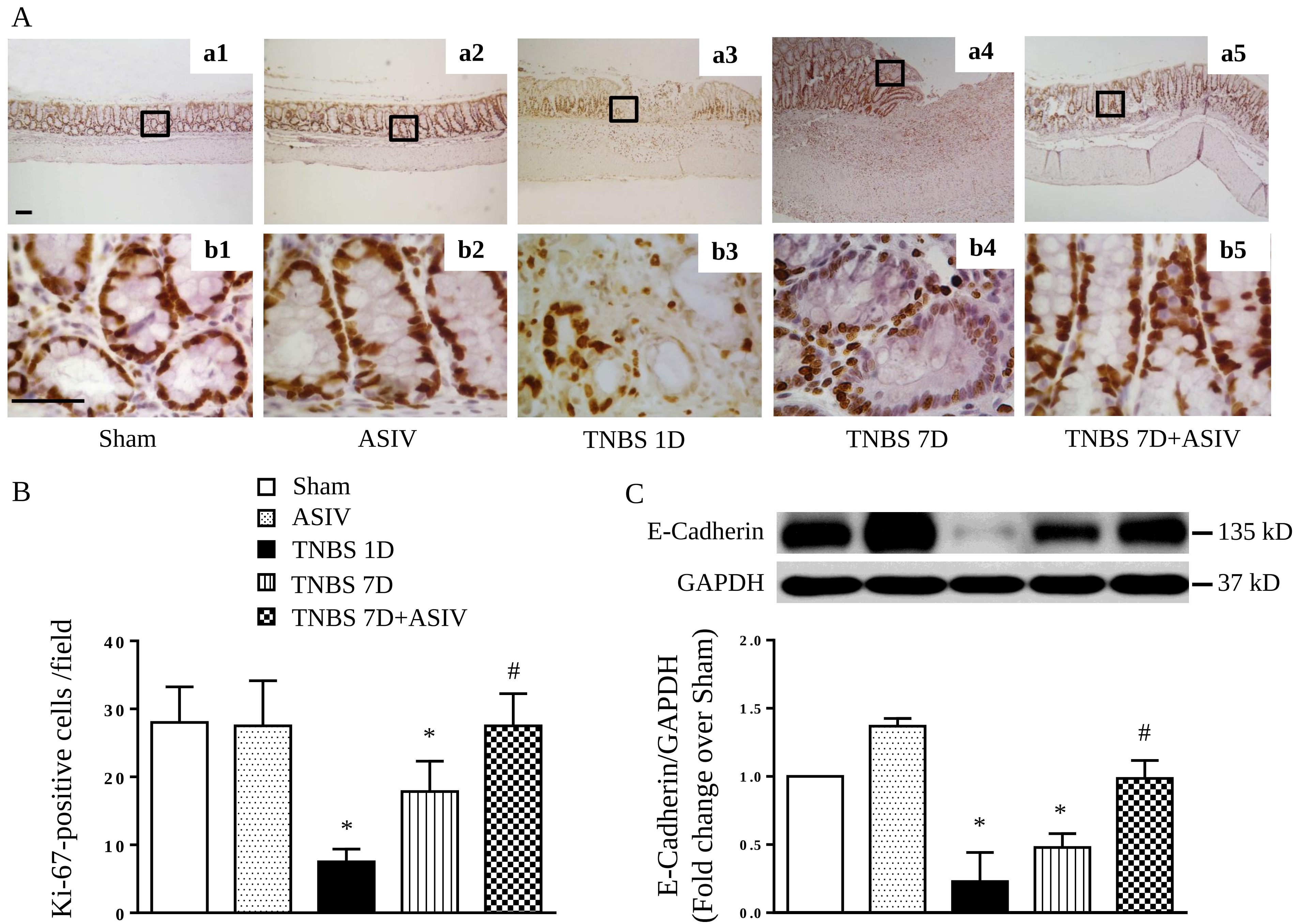

Supplement Figure 4

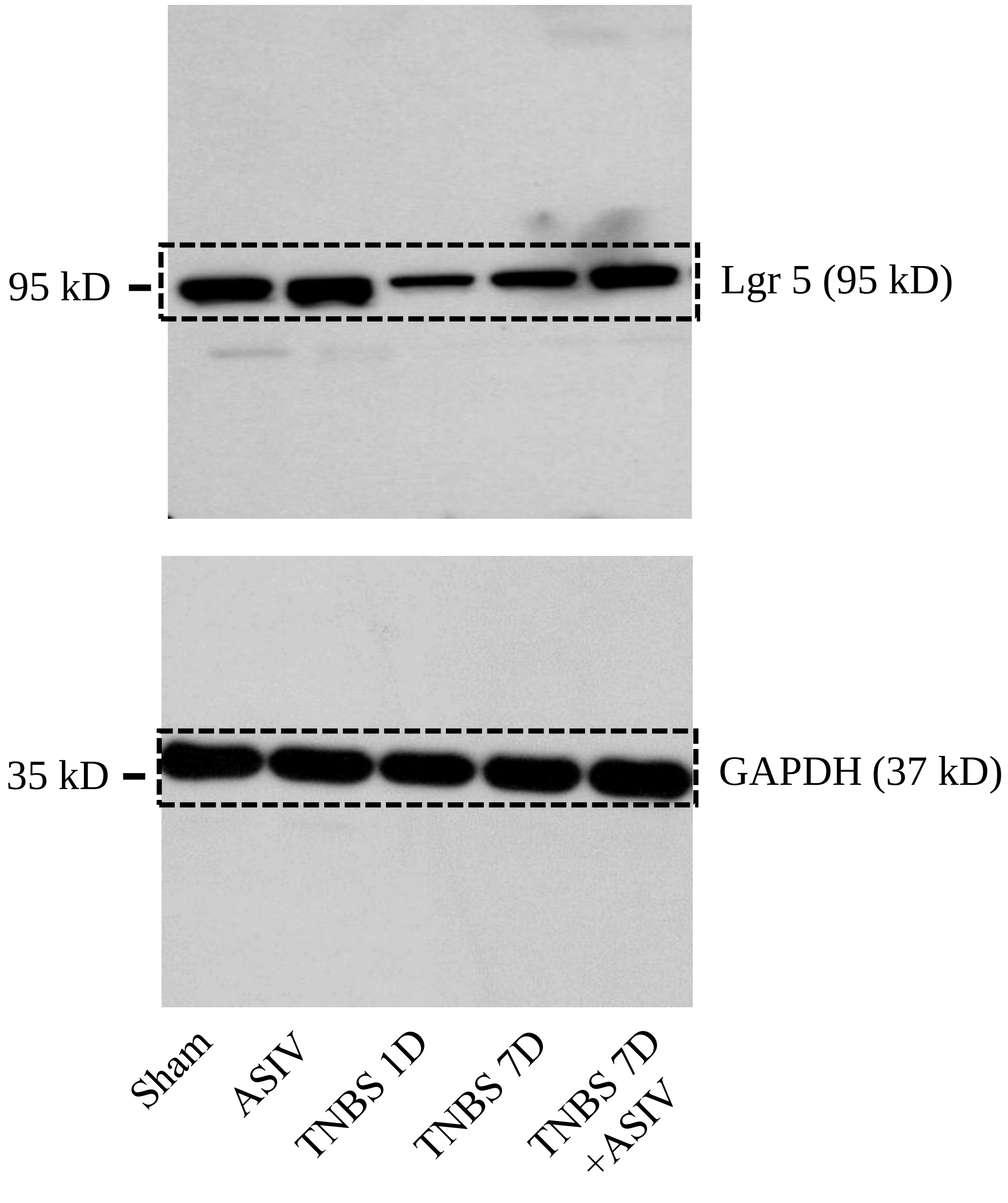

Supplement Figure 5

Fig. 5A

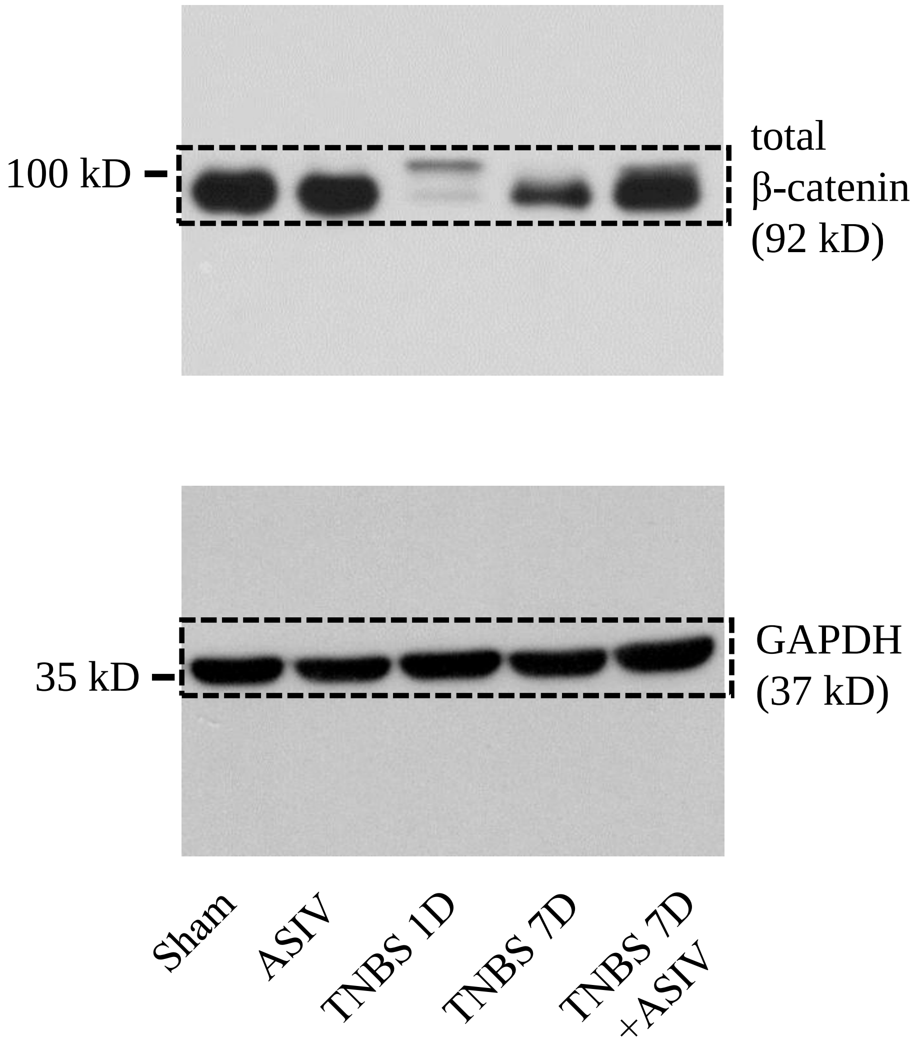

Fig. 5B

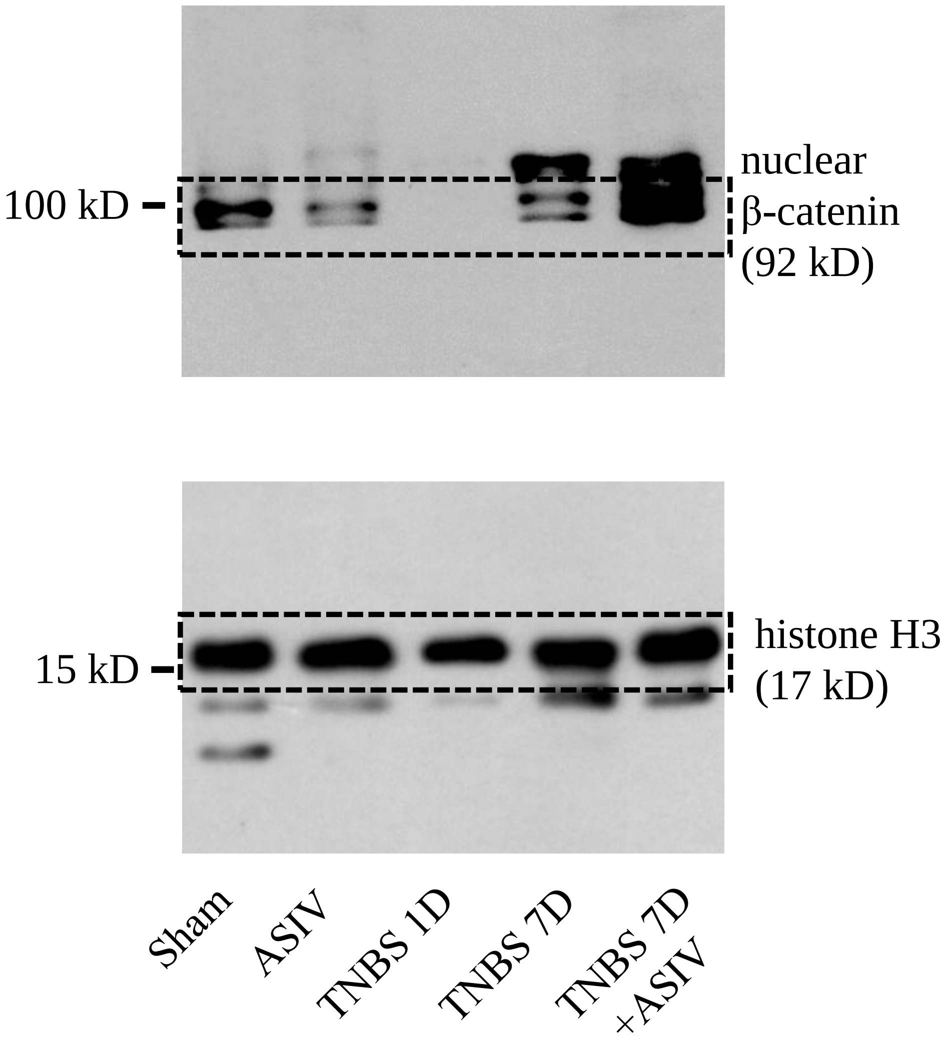

Supplement Figure 6

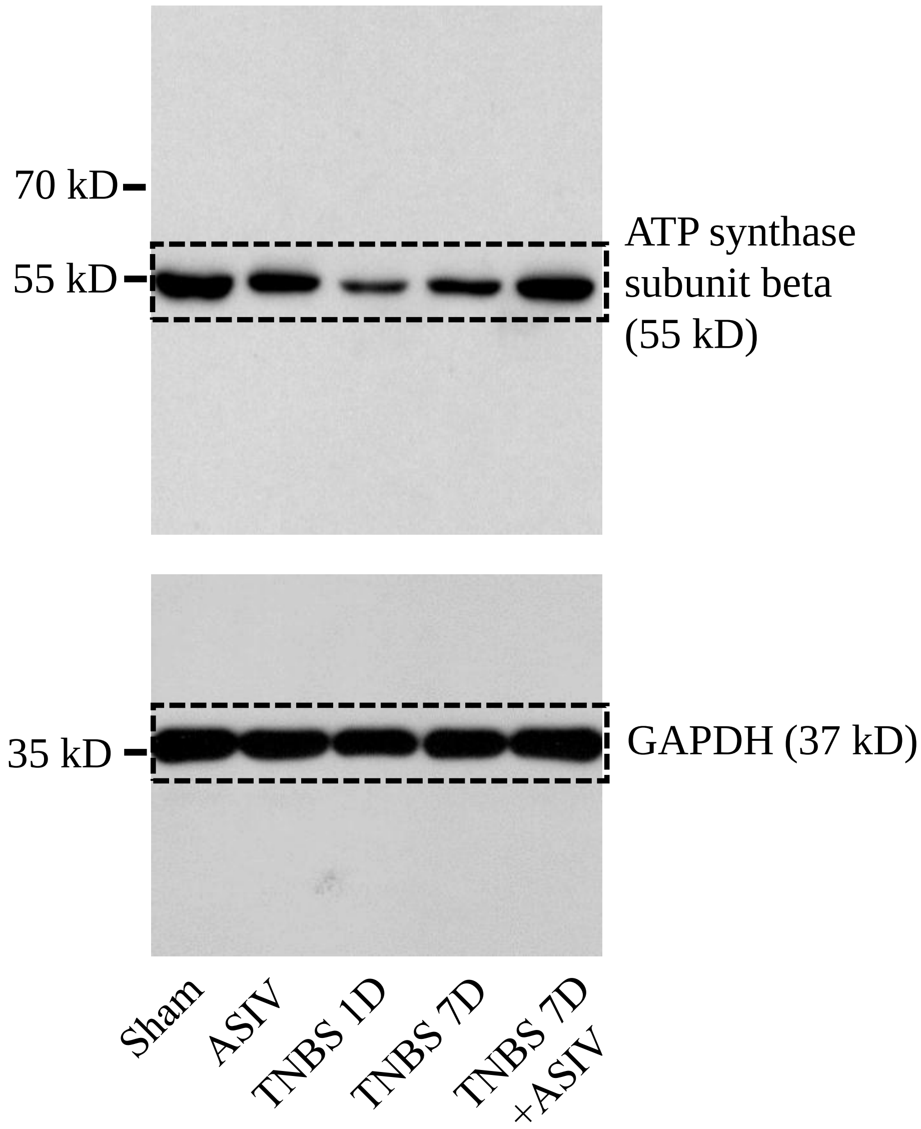

Supplement Figure 7

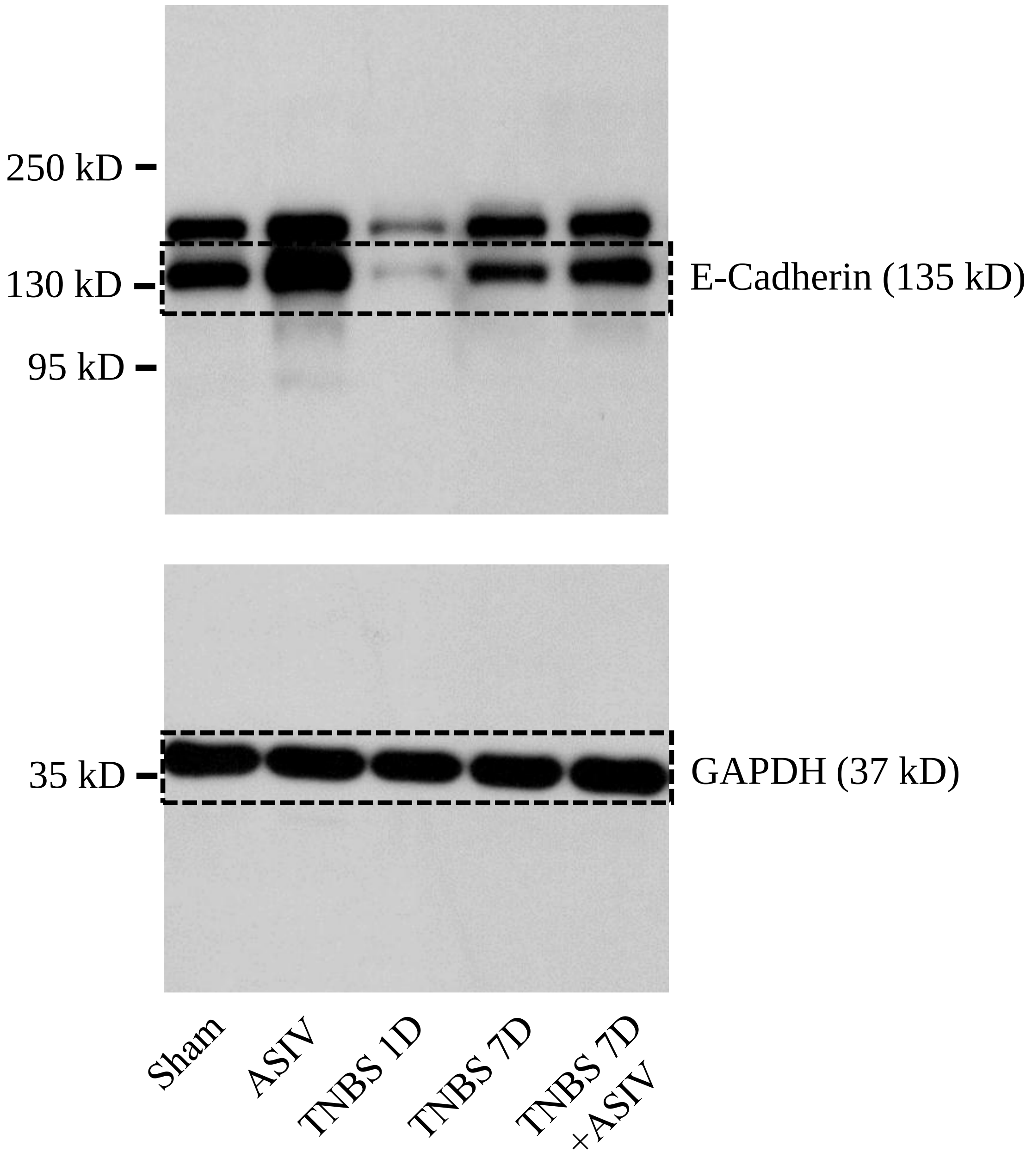

Supplement figure legend

Supplement Figure 1. ASIV reduces TNBS-induced colitis in rats in a dose- and time-dependent manner. (A) Representative image of colon in different groups. (B) Macroscopic injury score. (C) Colon length. (D) Colon weight index. Sham: sham group; ASIV: ASIV (10 mg/kg) alone group; TNBS 1D: TNBS treatment for 1 day group; TNBS 4D: TNBS treatment for 1 days followed by saline treatment for 3 days group; TNBS 4D+ASIV 5: TNBS treatment for 1 day followed by ASIV (5 mg/kg) treatment for 3 days group; TNBS 4D+ASIV 10: TNBS treatment for 1 day followed by ASIV (10 mg/kg) treatment for 3 days group; TNBS 7D: TNBS treatment for 1 days followed by saline treatment for 6 days group; TNBS 7D+ASIV 5: TNBS treatment for 1 day followed by ASIV (5 mg/kg) treatment for 6 days group; TNBS 7D+ASIV 10: TNBS treatment for 1 day followed by ASIV (10 mg/kg) treatment for 6 days group. Data are mean ± SEM (N=8). * $p < 0.05$ vs. Sham group, # $p < 0.05$ vs. TNBS 7D group.

Supplement Figure 2. ASIV suppresses inflammatory cell infiltration in TNBS-induced colitis in rats. (A) Representative images of immunohistochemistry of the tissues from different groups with double staining for MPO (brown) and CD68 (red) (a1-a5). Bar = 100 μm. The area within the rectangle in each picture is enlarged and presented below, correspondingly, to display the mucosa (b1-b5) and submucosa (c1-c5) in each group. Bar = 10 μm. (B) Quantification analysis of MPO-positive cells in different groups. (C) Quantification analysis of CD68-positive cells in different groups. Sham: sham group; ASIV: ASIV alone group; TNBS 1D: TNBS treatment for 1 day group; TNBS 7D: TNBS treatment for 1 days followed by saline treatment for 6 days group; TNBS 7D+ASIV: TNBS treatment for 1 day followed by ASIV treatment for 6 days group. Data are mean ± SEM (N=8). * $p < 0.05$ vs. Sham group, # $p < 0.05$ vs. TNBS 7D group.

Supplement Figure 3. ASIV promotes epithelial cell regeneration in TNBS-induced colitis in rats. (A) Representative images of immunohistochemistry staining for Ki67 (brown) of colonic tissues from different groups (a1-a5). Bar = 100 μm. The area within

the rectangle in each picture is enlarged and presented below correspondingly (b1-b5). Bar = 50 μm. (B) Quantification analysis of Ki67-positive cells in different groups (N=8). (C) Representative Western blots and statistical analysis of E-Cadherin in colon tissues from different groups (N=5). Sham: sham group; ASIV: ASIV alone group; TNBS 1D: TNBS treatment for 1 day group; TNBS 7D: TNBS treatment for 1 days followed by saline treatment for 6 days group; TNBS 7D+ASIV: TNBS treatment for 1 day followed by ASIV treatment for 6 days group. Data are mean ± SEM. * $p < 0.05$ vs. Sham group, # $p < 0.05$ vs. TNBS 7D group.

Supplement Figure 4. Source data for Figure 4C. Sham: sham group; ASIV: ASIV alone group; TNBS 1D: TNBS treatment for 1 day group; TNBS 7D: TNBS treatment for 1 days followed by saline treatment for 6 days group; TNBS 7D+ASIV: TNBS treatment for 1 day followed by ASIV treatment for 6 days group.

Supplement Figure 5. Source data for Figure 5A and B. Sham: sham group; ASIV: ASIV alone group; TNBS 1D: TNBS treatment for 1 day group; TNBS 7D: TNBS treatment for 1 days followed by saline treatment for 6 days group; TNBS 7D+ASIV: TNBS treatment for 1 day followed by ASIV treatment for 6 days group.

Supplement Figure 6. Source data for Figure 8D. Sham: sham group; ASIV: ASIV alone group; TNBS 1D: TNBS treatment for 1 day group; TNBS 7D: TNBS treatment for 1 days followed by saline treatment for 6 days group; TNBS 7D+ASIV: TNBS treatment for 1 day followed by ASIV treatment for 6 days group.

Supplement Figure 7. Source data for supplement Figure 3C. Sham: sham group; ASIV: ASIV alone group; TNBS 1D: TNBS treatment for 1 day group; TNBS 7D: TNBS treatment for 1 days followed by saline treatment for 6 days group; TNBS 7D+ASIV: TNBS treatment for 1 day followed by ASIV treatment for 6 days group.
